# A Descriptive Analysis of Nasal Polyposis in HIV Positive Versus HIV Negative Patients

**DOI:** 10.1007/s12070-024-04674-z

**Published:** 2024-04-10

**Authors:** Thobile Molokomme, Shivesh Maharaj, Shahpar Motakef

**Affiliations:** School of Clinical Medicine, Faculty of Health Sciences, University of the Witwatersrand, Johannesburg, South Africa

**Keywords:** Nasal polyps, HIV, Pathophysiology nasal polyposis

## Abstract

Nasal polyposis (NP) represents a benign proliferation of soft tissue tumors within the nasal cavity and paranasal sinuses, characterized by chronic inflammation of the sinonasal mucosa. This phenomenon, attributed to various environmental and physiological factors, presents clinically as semi-transparent masses with variable morphology, often obstructing nasal passages and causing respiratory compromise, olfactory dysfunction, and recurrent infections. Predominantly associated with chronic rhinosinusitis (CRS), NP poses significant challenges in diagnosis and management, particularly in the context of comorbid conditions such as human immunodeficiency virus (HIV) infection. HIV infection, known for its debilitating effects on the immune system, is theorized to exacerbate NP development and manifestation through mechanisms involving CD4 cell depletion and dysregulation of immune responses. Despite extensive research, elucidating potential pathways linking HIV infection to NP, comprehensive understanding remains elusive. This study aims to address this knowledge gap by conducting a retrospective chart review of patients presenting with NP at Charlotte Maxeke Johannesburg Academic Hospital between January 2016 and December 2020. The primary objective is to investigate the influence of HIV status on the clinical, radiological, and histological features of NP. Data collection, encompassing patient demographics, HIV status, clinical presentations, radiological findings, and histopathological characteristics, will be conducted between March 2021 and August 2022. Preliminary analysis of collected data reveals a cohort of 41 patients meeting inclusion criteria, with notable exclusions based on undisclosed HIV status and incomplete documentation. Initial findings suggest a nuanced interplay between genetic predisposition, environmental factors, and HIV status in NP pathogenesis, underscoring the need for further research to validate these observations. In conclusion, this study underscores the importance of elucidating the complex relationship between HIV infection and NP to optimize diagnostic and therapeutic approaches, particularly in regions with a high HIV prevalence such as South Africa. By comprehensively assessing the clinical, radiological, and histological features of NP in HIV-positive and HIV-negative populations, this research endeavours to enhance our understanding of NP pathophysiology and improve patient outcomes.

## Introduction

### Background on Nasal Polyposis and HIV Infection

Nasal polyposis (NP) denotes the benign proliferation of soft tissue tumors within the nasal cavity and sinuses. These growths, varying in size and morphology, often manifest as semi-transparent masses resembling grapes or teardrops [5]. While smaller polyps may remain asymptomatic, larger formations can impede nasal passages, leading to respiratory complications, olfactory dysfunction, and recurrent infections. NP arises from chronic inflammation of the sinonasal mucosa, attributed to diverse environmental and physiological triggers including allergies, asthma, and aspirin sensitivity [12]. Predominantly found in individuals with chronic rhinosinusitis (CRS), NP typically emerges from the middle meatus and ethmoidal regions, thus often conflated with CRS with nasal polyposis (CRSwNP).

Human immunodeficiency virus (HIV) infection precipitates immune system compromise, particularly by targeting CD4 cells, diminishing the body's ability to combat infections [23]. Coincidentally, the association between NP and HIV infection suggests a plausible correlation rooted in immunological dysfunction, among other pathways elucidated in extant literature [6, 10, 28, 35].

### Prevalence of HIV and Nasal Polyposis in the World and South Africa

Globally, the burden of HIV remains significant, with an estimated 38.4 million individuals living with HIV and 1.5 million new infections reported in 2021 [34]. Sub-Saharan Africa bears the brunt of this epidemic, with approximately 1 in 25 adults infected. South Africa, in particular, harbors a substantial portion of the global HIV burden, with approximately 8.2 million individuals living with HIV in 2021, representing nearly 14% of the national population [32]. Despite strides in treatment and testing, a significant proportion of HIV-positive individuals remain undiagnosed or disengaged from care, predisposing them to HIV-related morbidities, including sinonasal complications.

### Importance of Understanding Clinical, Radiological, and Histological Features of Nasal Polyposis in HIV-Positive Versus HIV-Negative Patients

The rising incidence of NP worldwide prompts the imperative to elucidate its etiology, particularly within the context of South Africa, where data on risk factors and treatment outcomes remain scarce [19]. Presently, guidelines such as the European Position Paper on Rhinosinusitis (EPOS) provide recommendations on NP management, yet overlook the unique challenges posed by the HIV epidemic [8]. In South Africa, the nexus between HIV and NP warrants exploration [14, 26], considering the nation's historical mining activities and concomitant environmental health hazards [18]. Given the potential convergence of HIV-associated immunodeficiency and environmental factors predisposing individuals to NP, investigating the clinical, radiological, and histological characteristics of NP in HIV-positive versus HIV-negative cohorts is paramount. Such endeavours aim to enhance NP management strategies and improve overall quality of life within the South African population.

## Methods

### Study Design

A retrospective chart review methodology was employed, utilizing pre-recorded or historical patient data extracted from medical records as the primary data source [21]. The study period spanned from January 1, 2016, to December 31, 2020, focusing on patients aged 18 and older diagnosed with nasal polyposis who presented at Charlotte Maxeke Johannesburg Academic Hospital (CMJAH).

#### Primary Exposure and Outcome

The primary exposure of interest was HIV status, aiming to elucidate its role in the development and clinical manifestation of nasal polyps.

#### Inclusion Criteria: Patients Meeting the Following Criteria were Included in the Study

- Aged 18 and older
- Diagnosis of nasal polyps confirmed clinically and radiologically
- Histological confirmation of nasal polyposis
- Known HIV status

#### Exclusion Criteria

Patients younger than 18 years and those with unknown HIV status were excluded from the study.

#### Sampling

Convenience sampling was employed, encompassing all eligible patient records within the specified study period [31].

#### Sample Size Calculation

A sample size of 41 records was ultimately reviewed, determined based on the recommendation of at least 5 events per variable in a retrospective chart review, considering six main predictor variables [29].

### Study Setting

The study was conducted at the Department of Otorhinolaryngology at Charlotte Maxeke Johannesburg Academic Hospital (CMJAH), a tertiary healthcare facility with 1088 beds, offering specialized services and serving as a teaching institution for the University of the Witwatersrand Health Sciences.

### Study Population

The study population comprised individuals aged 18 and older who presented to the CMJAH Department of Otorhinolaryngology between January 1, 2016, and December 31, 2020, with available clinical, radiological, and/or histological confirmation of nasal polyposis and HIV status.

### Data Collection

Patient files were accessed to retrieve clinical history, presentation, management, and radiological findings, supplemented by histology results obtained from the National Health Laboratory Service (NHLS) track. Data were recorded in a Microsoft Excel 2016 database, including demographic information and Lund McKay scores for radiological assessment.

### Statistical Data Analysis

The captured data were analyzed using SPSS version 25 software. Normality tests were conducted for continuous variables, with descriptive statistics employed for summarization. Chi-square tests and independent samples t-tests were utilized for assessing associations and differences between categorical and continuous variables, respectively.

### Ethical Considerations

Ethical approval was obtained from the hospital CEO and the Ethics Committee at the University of Witwatersrand. Patient consent was not required due to the retrospective nature of the study, however data anonymization was ensured to safeguard patient confidentiality and privacy.

## Results

**Fig. 1:**
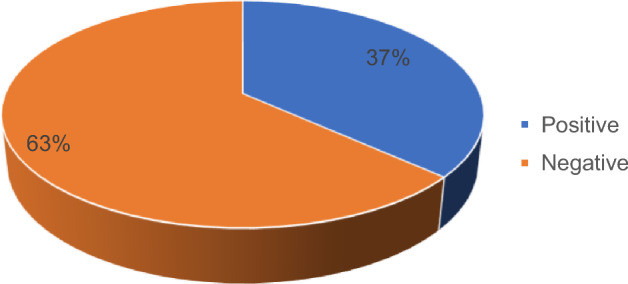
HIV status

Data collection occurred between March 2021 and August 2022 at the Department of Otorhinolaryngology at CMJAH. A total of 41 patient charts that met the inclusion criterion i.e. nasal polyps with a clinical, radiological and/or histological confirmation were retrieved from the hospital system. Upon further assessment, 10 of the total charts were excluded from enrolment into the study as they had an unknown HIV status, and 1 was excluded because it did not have the age documented. The study requirement was that participants should have been 18 and older and a chart with no age meant that it could not be established whether they qualified to be in the study sample or not. Resultantly, a total of 30 patients were analysed for this study. About 63% of the participants were HIV-negative while 37% were living with HIV (Fig. 1). Table 1 presents demographic and clinical characteristics with the chi-square test results of the study sample in detail. The study was on average 40.40 ± 13.703 years old, and when HIV status was considered, patients living with HIV were a bit older than HIV-negative people. Eighteen (60%) participants were men. As far as gender is concerned, men accounted for 68.4% of the participants who were HIV-negative whereas women accounted for a higher proportion (54.5%) of those living with HIV. However, there was no evidence of a statistical difference between men and women regarding their HIV status (*p*-value = 0.266).

**Table 1:** Demographic and clinical characteristics of the sample by HIV Status

N (%)	Option	HIV STATUS			*P*-value
Variable		Positive (n = 11)	Negative (n = 19)	Total (n = 30)	
Gender	Male	5 (45.5%)	13 (68.4%)	18 (60%)	0.266
	Female	6 (54.5%)	6 (31.6%)	12 (40%)	
Nasal discharge	Yes	4 (36.4%)	6 (31.6%)	10 (33.3%)	1.000
	No	7 (63.6%)	13 (68.4%)	20 (66.7%)	
Nasal congestion	Yes	9 (81.8%)	16 (84.2%)	25 (83.3%)	1.000
	No	2 (18.2%)	3 (15.8%)	5 (16.7%)	
Facial pressure	Yes	3 (27.3%)	5 (26.3%)	8 (26.7%)	1.000
	No	8 (72.7%)	14 (73.7%)	22 (73.3%)	
Anosmia	Yes	4 (36.4%)	8 (42.1%)	12 (40%)	1.000
	No	7 (63.6%)	11 (57.9%)	18 (60%)	
Postnasal drip	Yes	0 (0%)	3 (15.8%)	3 (10%)	0.279
	No	11 (100%)	16 (84.2%)	27 (90%)	
Ageusia	No	11 (100%)	19 (100%)	30 (100%)	–
Cough	No	10 (100%)	8 (100%)	18 (100%)	–
Sneezing	Yes	0 (0%)	4 (21.1%)	4 (13.3%)	0.268
	No	11 (100%)	15 (78.9%)	26 (86.7%)	
Asthma	Yes	2 (18.2%)	3 (15.8%)	5 (16.7%)	1.000
	No	9 (81.8%)	16 (84.2%)	25 (83.3%)	
Aspirin sensitivity	Yes	1 (9.1%)	2 (10.5%)	3 (10%)	1.000
	No	10 (90.9%)	17 (89.5%)	27 (90%)	
Age (mean ± Standard Deviation)		44.45 ± 13.344	38.05 ± 30.701	40.40 ± 13.703	0.224
Lund McKay (radiological (mean ± Standard Deviation)		31.00 ± 13.682	31.95 ± 12.177	31.60 ± 12.522	0.846

When clinical symptoms were taken into consideration, more than 33% of the total sample presented to the study site with a nasal discharge and when stratified by HIV status, nasal discharge was slightly more prevalent in the group living with HIV (36.4) than in the HIV-negative group (31.4%). Again, there was no indication of statistical differences between these groups (*p*-value = 1.000). Nasal congestion was noted in 83.3% of participants and was highly prevalent in both HIV-negative patients (84.2%) and patients living with HIV (81.8%) with no significant difference between the groups (*p*-value = 1.000). Approximately 40% had anosmia and it was more prevalent in the HIV-negative group (42.1%) than their counterparts living with HIV (36.4%). Furthermore, facial pressure was found in less than 27% of the sample and its prevalence was similar in both HIV status groups. Postnasal drip and aspirin sensitivity accounted for only 10% of clinical symptoms noted in the sample, separately, and only HIV-negative patients had the former. Nonetheless, there was no indication that there was a statistical difference between HIV-negative and HIV-positive patients. For aspirin sensitivity, 10.5% of the HIV-negative people had it and 9.1% of those living with HIV did too. Again, no statistical evidence of difference was noted between groups. In addition, the prevalence of asthma was 16.7% in the total sample. When stratified by HIV status, people living with HIV had a slightly more prevalence (18.2%) compared to their HIV-negative counterparts who had an asthma prevalence of 15.8%. What is also noteworthy on the clinical symptoms’ front is that no patient presented with either cough or ageusia. Finally, the Lund McKay Score was on average 31.6 with a standard deviation of 12.5 and patients who are HIV-negative had a higher Lund McKay score of 31.9 on average while patients living with HIV had an average score of 31.0.

A chi-square test of association was conducted to assess whether there was an association between categorical variables and the HIV status of the patients. Independent samples t-test was conducted for the two continuous variables namely, age and Lund McKay (radiological). The results are presented below.

Table 2 presents genetic factors associated with nasal polyps by HIV status. None of the patients had either ciliary dyskinesia, cystic fibrosis or Kartegener syndrome. However, 20% were reported to have atopy. When HIV status was accounted for in atopy, 21.1% of HIV-negative had it while 18.2% of patients living with HIV had it. Nevertheless, no statistically significant difference was noted between the two groups (*p*-value = 1.000). Sneezing was prevalent in about 6.7% of the general sample and only HIV-negative patients reported it although there was no evidence of statistical difference by HIV status. Similarly, an itchy nose was reported in 6.7% of the total patients and no one living with HIV had it. However, there were no notable statistical differences between HIV-negative patients and those living with HIV as the p-value was greater than 0.005.

**Table 2:** Genetic factors

n (%)	Option	HIV STATUS			*P*-value
Variable		Positive (n = 11)	Negative (n = 19)	Total (n = 30)	
Genetic factors	No	11 (100%)	19 (100%)	30 (100%)	–
Ciliary dyskinesia	No	11 (100%)	19 (100%)	30 (100%)	–
Cystic fibrosis	No	11 (100%)	19 (100%)	30 (100%)	–
Kartegener syndrome	No	11 (100%)	19 (100%)	30 (100%)	–
Atopy	Yes	2 (18.2%)	4 (21.1%)	6 (20%)	1.000
	No	9 (81.8%)	15 (78.9%)	24 (80%)	
Sneezing	Yes	0 (0%)	2 (10.5%)	2 (6.7%)	0.520
	No	11 (100%)	17 (89.5%)	28 (93.3%)	
Itchy nose	Yes	0 (0%)	2 (10.5%)	2 (6.7%)	0.520
	No	11 (100%)	17 (89.5%)	28 (93.3%)	

Table 3 shows histological factors observed in the charts. A total of 20 (66.7%) had respiratory mucosa. This was notably more prevalent in patients living with HIV (72.7%) than HIV-negative patients (63.2%). Subepithelial edema hyperplasia accounted for 50% of the total sample and was most commonly encountered in HIV-negative people (63.2%) compared to people living with HIV (27.3%). But there was no significant difference noted between the two HIV status groups. Over 73% of the general sample reportedly had lymphocytes and people living with HIV had a higher proportion (81.8%) of lymphocytes reported than their HIV-negative counterparts (68.4%). Further, mucoserous glands were found in 33.3% of the total sample but HIV-negative patients had an even higher proportion (42.1%) than the general sample and patients living with HIV (18.2%). Notwithstanding that, there were group statistical differences noted as was the case with lymphocytes too. Eosinophils +  +  + was present in 20% of the total participants and was slightly more prevalent in the HIV-negative group than the HIV-positive one though with no notable statistical difference (*p*-value = 1.000).

**Table 3:** Histology

N (%)	Option	HIV STATUS			*P*-value
Variable		Positive (n = 11)	Negative (n = 19)	Total (n = 30)	
Respiratory mucosa	Yes	8 (72.7%)	12 (63.2%)	20 (66.7%)	0.702
	No	3 (27.3%)	7 (36.8%)	10 (33.3%)	
Subepithelial oedema hyperplasia	Yes	3 (27.3%)	12 (63.2%)	15 (50%)	0.128
	No	8 (72.7%)	7 (36.8%)	15 (50%)	
Lymphocytes	Yes	9 (81.8%)	13 (68.4%)	22 (73.3%)	0.672
	No	2 (18.2%)	6 (31.6%)	8 (26.7%)	
Mucoserous glands	Yes	2 (18.2%)	8 (42.1%)	10 (33.3%)	0.246
	No	9 (81.8%)	11 (57.9%)	20 (66.7%)	
Eosinophils + + +	Yes	2 (18.2%)	4 (21.1%)	6 (20%)	1.000
	No	9 (81.8%)	15 (78.9%)	24 (80%)	
Plasma cells	Yes	8 (72.7%)	12 (63.2%)	20 (66.7%)	0.702
	No	3 (27.3%)	7 (36.8%)	10 (33.3%)	
Eosinophils	Yes	7 (63.6%)	13 (68.4%)	20 (66.7%)	1.000
	No	4 (36.4%)	6 (31.6%)	10 (33.3%)	
Basement membrane thickening	Yes	5 (45.5%)	2 (10.5%)	7 (23.3%)	0.068
	No	6 (54.5%)	17 (89.5%)	23 (76.7%)	
Neutrophils	Yes	1 (9.1%)	2 (10.5%)	3 (10%)	1.000
	No	10 (90.9%)	17 (89.5%)	27 (90%)	
Histiocytes	Yes	1 (9.1%)	0 (0%)	1 (3.3%)	0.367
	No	10 (90.9%)	19 (100%)	29 (96.7%)	
Subepithelial oedema hyperplasia	Yes	2 (18.2%)	5 (26.3%)	7 (23.3%)	1.000
	No	9 (81.8%)	14 (73.7%)	23 (76.7%)	
Stratified squamous	Yes	2 (18.2%)	1 (5.3%)	3 (10%)	0.537
	No	9 (81.8%)	18 (94.7%)	27 (90%)	
Squamous metaplasia	Yes	1 (9.1%)	3 (15.8%)	4 (13.3%)	1.000
	No	10 (90.9%)	16 (84.2%)	26 (86.7%)	
Goblet cells	Yes	0 (0%)	2 (10.5%)	2 (6.7%)	0.520
	No	11 (100%)	17 (89.5%)	28 (93.3%)	
Ulcerations	Yes	0 (0%)	2 (10.5%)	2 (6.7%)	0.520
	No	11 (100%)	17 (89.5%)	28 (93.3%)	
Fibrosis	Yes	1 (9.1%)	1 (5.3%)	2 (6.7%)	1.000
	No	10 (90.9%)	18 (94.7%)	28 (93.3%)	
Pseudostratified columnar	No	11 (100%)	19 (100%)	30 (100%)	–
Pseudostratified columnar ciliated	Yes	1 (9.1%)	1 (5.3%)	2 (6.7%)	1.000
	No	10 (90.9%)	18 (94.7%)	28 (93.3%)	
Pseudostratified epithelium columnar	Yes	0 (0%)	3 (15.8%)	3 (10%)	0.279
	No	11 (100%)	16 (84.2%)	27 (90%)	
Squamous epithelium {C itation}	Yes	0 (0%)	1 (5.3%)	1 (3.3%)	1.000
	No	11 (100%)	18 (94.7%)	29 (96.7%)	
Stratified squamous mucosa	No	11 (100%)	19 (100%)	30 (100%)	–
Charcot Leyden crystals	Yes	0 (0%)	1 (5.3%)	1 (3.3%)	1.000
	No	11 (100%)	18 (94.7%)	29 (96.7%)	
Cuboidal mucosa	Yes	0 (0%)	1 (5.3%)	1 (3.3%)	1.000
	No	11 (100%)	18 (94.7%)	29 (96.7%)	
Charcot Leyden crystals	No	11 (100%)	19 (100%)	30 (100%)	–
Bone overall degree of inflammation	Yes	1 (9.1%)	0 (0%)	1 (3.3%)	0.367
	No	10 (90.9%)	19 (100%)	29 (96.7%)	
Allergic mucin	Yes	0 (0%)	1 (5.3%)	1 (3.3%)	1.000
	No	11 (100%)	18 (94.7%)	29 (96.7%)	
Degranulated eosinophilia aggregates	Yes	0 (0%)	1 (5.3%)	1 (3.3%)	1.000
	No	11 (100%)	18 (94.7%)	29 (96.7%)	
Pseudostratified goblet	Yes	0 (0%)	1 (5.3%)	1 (3.3%)	1.000
	No	11 (100%)	18 (94.7%)	29 (96.7%)	

Furthermore, plasma cells were present in about 67% and 73% of the general sample and HIV-positive group, respectively. Regardless, plasma cells were reported in 63% of the HIV-negative patients and there seemed to be no significant difference between HIV status groups (*p*-value = 0.702). Like plasma cells, eosinophils had a prevalence of 66.7%.However, the prevalence between groups was not that different from each as patients living with HIV had a 63.6% prevalence and HIV-negative people had an eosinophils prevalence of 68.4%. 33.3% of the total patients had a basement membrane thickening and this was disproportionately higher in the HIV-positive group (45.5%) compared to the HIV-negative one (10.5%). Interestingly, the p-value was 0.068 suggesting marginal difference between groups. Neutrophils were encountered in 10% of the general sample, 10.5% in the HIV-negative group and 9.1% in patients living with HIV and there were no indications of any significant difference between the two groups. Histiocytes were not that common as it was only evident in 3.3% in the total sample. They, however, were only observed in patients living with HIV although the chi-square test was not statistically significant. Stratified squamous and squamous metaplasia accounted for 10% and 13.3% in the general sample, respectively. But when HIV status was considered, 18.2% of patients living with HIV had stratified squamous while only 5.3% had it in the HIV-negative group. Inversely, squamous metaplasia was higher in HIV-negative people (15.8%) than in those living with HIV (9.1%). In both instances, no statistically significant difference was noted though. Of all participants, 6.7% had goblet cells, and ulcerations and they were only present in HIV-negative patients, however this was no indication that there was a difference between groups (*p*-value = 0.520). Similarly, 6.7% of everyone had fibrosis and pseudostratified ciliated columnar, separately. Both conditions were present in 9.1% of people with HIV and in 5.3% of their negative peers. Again, no significant difference was found between the groups. Notably, no patient had pseudostratified columnar.

However, 10% and 15.8% of the total sample and HIV-negative patients had pseudostratified columnar epithelial, respectively.

Squamous epithelium stratified squamous mucosa, cuboidal mucosa, allergic mucin, pseudostratified goblet, degranulated eosinophilia aggregates and Charcot Leyden crystals were independently observed in 3.3% of the sample, and 5.3% of those who are HIV-negative. No HIV-positive patient had any of these, and no statistical difference was found. Bone overall degree of inflammation was also noted in 3.3% of the total study participants. However, it only affected patients living with HIV (9.1%) although no statistically significant difference between those with HIV and those without.

## Discussion

The current study investigated the differences in clinical, radiological, and histological features of nasal polyps between HIV-positive and HIV-negative patients and found that there was no statistical difference between these patients.

Interestingly, the prevalence of nasal discharge, nasal congestion, anosmia, facial pressure, postnasal drip, and aspirin sensitivity were comparable between both HIV-positive and HIV-negative patients. There was no significant difference in the prevalence of these clinical symptoms between the two groups. There are no studies that point to marked differences between the groups regarding these symptoms. That is because most studies on nasal polyps have been conducted in low HIV prevalence regions and thus, have not considered it in the process [1, 11, 13].

Despite this, this study found asthma was more prevalent in HIV-positive patients than HIV-negative patients, although the difference was not statistically significant. This finding aligns with literature, studies have found asthma to be highly prevalent in people living with HIV [15, 17, 27] Respiratory diseases are common in people with HIV resulting from severe immune dysregulation from the infection. It is still unclear how asthma and HIV relate however, the IgE and cytokine network are implicated [17].

Moreover, no patient presented with either cough or ageusia. These findings suggest that the presence of nasal polyps may not be significantly affected by HIV status.

Additionally, the Lund McKay Score was assessed in both HIV-positive and HIV-negative patients, and it was found that HIV-negative patients had a slightly higher score on average compared to HIV-positive patients. Although this difference was not statistically significant, it indicates that HIV status may not affect the severity of nasal polyps. Again, no studies exist that indicate differences in scores.

Genetic factors associated with nasal polyps are important to consider, especially in patients living with HIV. In this study, none of the patients had ciliary dyskinesia, cystic fibrosis or Kartegener syndrome, which are known genetic factors associated with nasal polyps. However, atopy was reported in 20% of the sample. Although there was no statistically significant difference in atopy between HIV-negative and HIV-positive patients, it is still an important factor to consider in patients with nasal polyps. Atopy has been previously associated with the development of nasal polyps, and it is believed to be a risk factor for the recurrence of nasal polyps after surgical treatment [25]. It is apparently common in South Africa due to environmental factors [20]. Therefore, its prevalence may be indicative of what is happening at the population level in the country. However, a study on rhinosinusitis and atopy among people living with HIV reported atopy being present in 18% of patients similar to what this study found [9]. However, the study in question was a case series and such studies generally rank low on the hierarchy of evidence due to their high susceptibility to bias. As such, it may be prudent to suggest that the result was only a fluke, especially in the absence of any more genetic evidence about HIV infection and atopy.

Furthermore, sneezing and itchy nose were reported in a small percentage of the total patients, with only HIV-negative patients reporting sneezing and no HIV-positive patients reporting itchy nose. These clinical symptoms could be associated with allergic rhinitis, which is a common comorbidity of nasal polyps. In patients living with HIV, the immune system is compromised, and this could affect their response to allergens and the development of allergic rhinitis [2, 3].

Additionally, results of this study provide insight into the histological factors associated with nasal polyps in HIV-positive and HIV-negative patients. The presence of respiratory mucosa was more common in patients living with HIV. This finding can be corroborated by a study by Sellers and colleagues [30] which found that the respiratory mucosa of HIV-infected patients showed decreased pro-inflammatory cytokine levels.

Basement membrane thickening was higher in the HIV-positive group, with a marginal difference noted. Particular cases of basement membrane disease in people living with HIV due to elevated levels of antibodies have been noted [22, 33]. This could explain the higher prevalence. Similarly, fibrosis and pseudostratified ciliated columnar were more prevalent in patients living with HIV. That is because immune activation and persistent inflammation are likely to contribute to collagen deposition and lymphoid tissue fibrosis in HIV-infected people [7].

Bone overall degree of inflammation was observed only in patients living with HIV. This result is in line with other research that suggested a link between HIV infection and bone inflammation. It has been hypothesized that HIV infection can result in chronic inflammation that causes bone loss, which may help persons living with HIV develop osteoporosis and increase their risk of fracture [16, 24]. It has been suggested that HIV-associated chronic inflammation may cause an increase in bone resorption and a decrease in bone formation, albeit the precise mechanism underlying this link is not entirely known. It is important to bear in mind that bone inflammation is a complex process, with HIV infection being only one of the numerous variables that might play a role in the development of this condition [4, 16, 24].

## Limitations

It is important to note that the current study has some limitations. Firstly, the sample size was relatively small, which may limit the generalizability of the findings. Secondly, the study only included patients from a single study site, which may limit the representativeness of the sample. Therefore, future studies with larger sample sizes and more diverse populations are necessary to further investigate the differences in nasal polyps between HIV-positive and HIV-negative patients.

## Conclusion

While there were some differences in clinical, radiological, and histological factors between HIV-positive and HIV-negative patients, there were no statistically significant differences in most cases. The findings suggest that the presence of nasal polyps may be influenced by a combination of genetic and environmental factors rather than HIV status alone. However, further research is needed to confirm these findings.
